# Intraarticular injection of microfragmented adipose tissue plus arthroscopy in isolated primary patellofemoral osteoarthritis is clinically effective and not affected by age, BMI, or stage of osteoarthritis

**DOI:** 10.1186/s10195-022-00628-9

**Published:** 2022-02-05

**Authors:** Michele Vasso, Katia Corona, Luigi Capasso, Giuseppe Toro, Alfredo Schiavone Panni

**Affiliations:** 1 Multidisciplinary Department of Medico-Surgical and Dentistry Specialties, “Luigi Vanvitelli” University of Campania, Naples, Italy; 2 Department of Medicine and Health Sciences “Vincenzo Tiberio”, University of Molise, Campobasso, Italy

**Keywords:** Adipose tissue, Adipose-derived stem cells, Patellofemoral osteoarthritis, Arthroscopic debridement, Knee

## Abstract

**Purpose:**

This study aimed to report the clinical and functional results of a series of patients with isolated primary patellofemoral osteoarthritis (PFOA) treated with intraarticular injection of microfragmented autologous adipose tissue plus knee arthroscopy. The results were also analyzed in relation to the age and body mas index (BMI) of patients, and to the stage of PFOA.

**Methods:**

Twenty-three patients with early-to-moderate (stage 1–3 according to the Iwano classification system) PFOA who received this treatment were retrospectively analyzed, with a mean follow-up of 22.1 ± 4.2 months. Patients were assessed using the International Knee Society (IKS) knee and function and visual analog scale (VAS) scores, and relative to their capacity for climbing stairs. Differences in improvements of IKS and VAS scores in relation to age (< 60 versus ≥ 60 years), BMI (< 30 versus ≥ 30 kg/m^2^), and stage of PFOA (stages 1–2 versus stage 3) were finally analyzed.

**Results:**

The mean IKS knee score significantly improved from 35.6 ± 14.9 points preoperatively to 61.9 ± 17.8 points at the latest follow-up, while the mean IKS function score significantly improved from 52.0 ± 14.7 points preoperatively to 82.3 ± 19.1 points at the latest follow-up. The mean VAS score significantly decreased from 8.7 ± 2.2 preoperatively to 5.2 ± 2.5 at the latest follow-up. A significant improvement in the capacity to climb stairs was found. No significant differences in improvements of IKS knee and function and VAS scores were found in relation to age, BMI, or stage of PFOA.

**Conclusion:**

Intraarticular injection of microfragmented autologous adipose tissue following arthroscopic debridement significantly improved overall clinical and functional scores in patients with early or moderate isolated primary PFOA at a mean follow-up of almost 2 years. Improvements were not significantly affected by age, BMI, or stage of PFOA.

**Level of evidence:**

Level IV, retrospective case series.

## Introduction

Patellofemoral osteoarthritis (PFOA) is involved in more than 45% of knee osteoarthritis (OA) cases, either isolated (half of those cases) or associated with femorotibial degeneration [24]. Most patients with isolated PFOA present primary or idiopathic PFOA, while a minority of patients present PFOA secondary to patellar instability or trauma [10, 37]. Females aged ≥ 50 years appear to have a higher prevalence of PFOA (41%) compared with males aged ≥ 50 years (23%) [25]. The management of isolated primary PFOA is still highly controversial, especially in patients with early or moderate patellofemoral degeneration. In these patients, results of conservative treatment [physiotherapy and/or taping, intraarticular injections with hyaluronic acid and/or platelet-rich plasma (PRP)] or nonreplacement surgery (arthroscopy, chondroplasty, lateral facetectomy) are often ineffective, while replacement surgery is certainly premature [37].

Intraarticular cell therapies with mesenchymal stem cells (MSCs) have been widely used in the past few years for managing knee OA [4, 12, 31]. MSCs are multipotent stromal cells with the possibility to differentiate into chondrocytes with adequate stimuli (growth factors and cytokines), therefore adapting to the microenvironment where they reside [29]. More recently, adipose tissue has become an important source of MSCs due to its abundance, ease of access, and simplicity of harvesting. Adipose tissue contains a greater concentration of MSCs (up to 2%) than bone marrow (0.02%) [16, 31]. Additionally, adipose-derived stem cells (ASCs) present a higher proliferation and chondrogenic potential than other human MSCs, and this potential is less affected by the age of the patient-donors [5, 7, 20]. It has been reported how intraarticular injection of autologous adipose tissue rich in ASCs in patients with knee OA increased glycosaminoglycan and type II collagen content in hyaline cartilage [16, 27], and resulted in radiological evidence of improved cartilage volume and confirmed hyaline-like cartilage on histology [22]. These findings were in line with observed improved VAS scores and clinical outcomes. Although several studies have already shown promising results in patients with knee OA treated with intraarticular injection of adipose tissue rich in ASCs (both previously expanded or microfragmented in the operating room) associated or not to arthroscopic debridement [1, 11, 15, 16, 21, 22, 26, 34], the use of this treatment is not yet supported by strong scientific evidence [17], especially regarding PFOA. To our knowledge, no previous studies have evaluated the effectiveness of adipose tissue knee injection in patients with isolated primary PFOA.

The aim of this study was, therefore, to report the clinical and functional results of a series of patients with isolated primary PFOA treated with intraarticular injection of autologous adipose tissue rich in ASCs obtained through a commercially available technique that intraoperatively provided microfragmented and minimally manipulated adipose tissue without cell expansion or enzymatic treatment. At the same time, all the patients underwent knee arthroscopy. Additionally, overall clinical and functional results were analyzed in relation to the age and BMI of patients, as well as the stage of PFOA.

## Materials and methods

From 2016 to 2020, 117 patients with generic knee OA underwent intraarticular injection of autologous microfragmented adipose tissue following knee arthroscopy. All operations were performed by two surgeons (M.V. and A.S.P.). The diagnosis of knee OA was made following the American College of Rheumatology criteria [30]. Selection criteria to perform this procedure were onset of symptoms of the index knee 6 or more months ago, and/or failure of the conservative treatment. Contraindications and exclusion criteria were recent trauma of the symptomatic knee with evident acute involvement of ligaments and/or menisci, infectious joint disease, chondromatosis or villonodular synovitis of the knee, malignancy, pregnancy, and patients on anticoagulant therapy or suffering from thrombocytopenia and/or coagulation disorder. Age and BMI were not considered a factor influencing the decision to use this protocol, although patients older than 80 years were generally excluded.

**Fig. 1:**
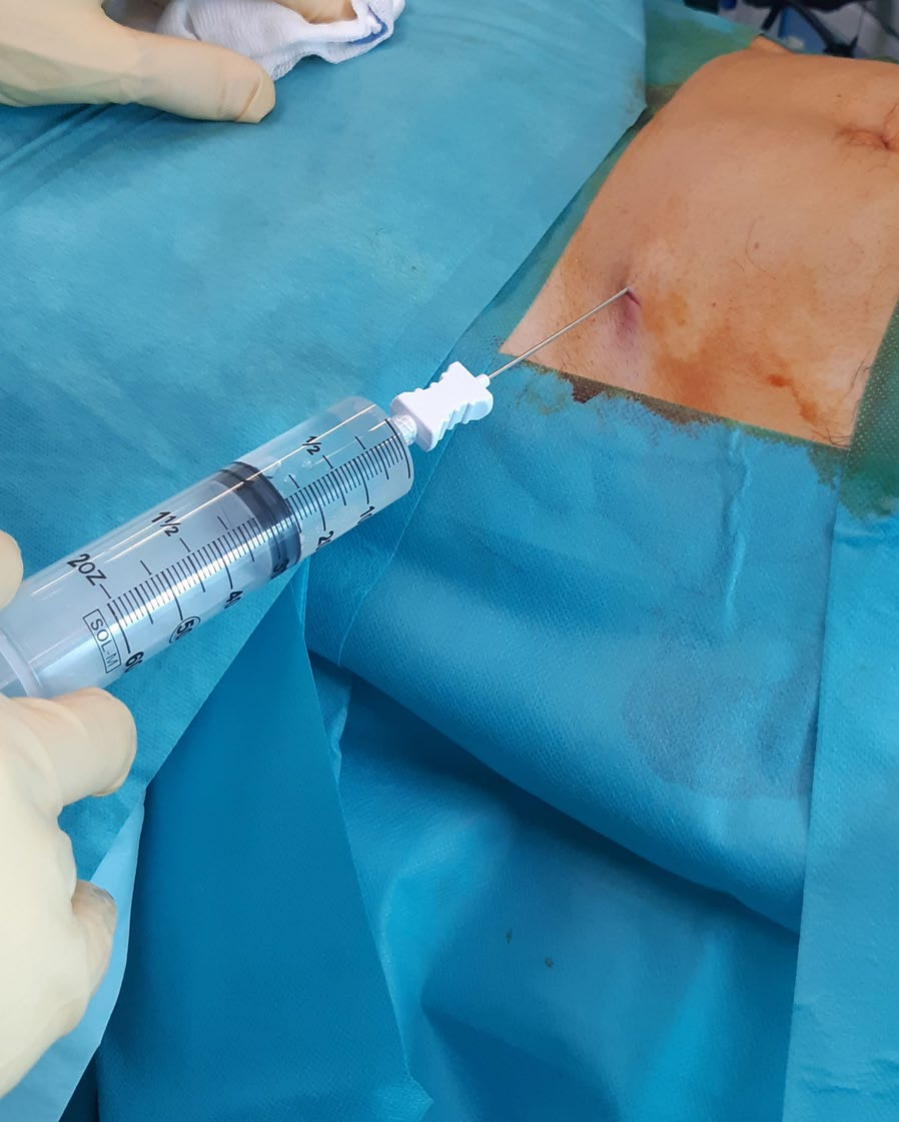
Saline solution mixed with ropivacaine and adrenaline (Klein solution) percutaneously injected into the subcutaneous tissue of the abdomen to expand the spaces inside of the adipose tissue and to allow successive lipoaspiration

**Fig. 2:**
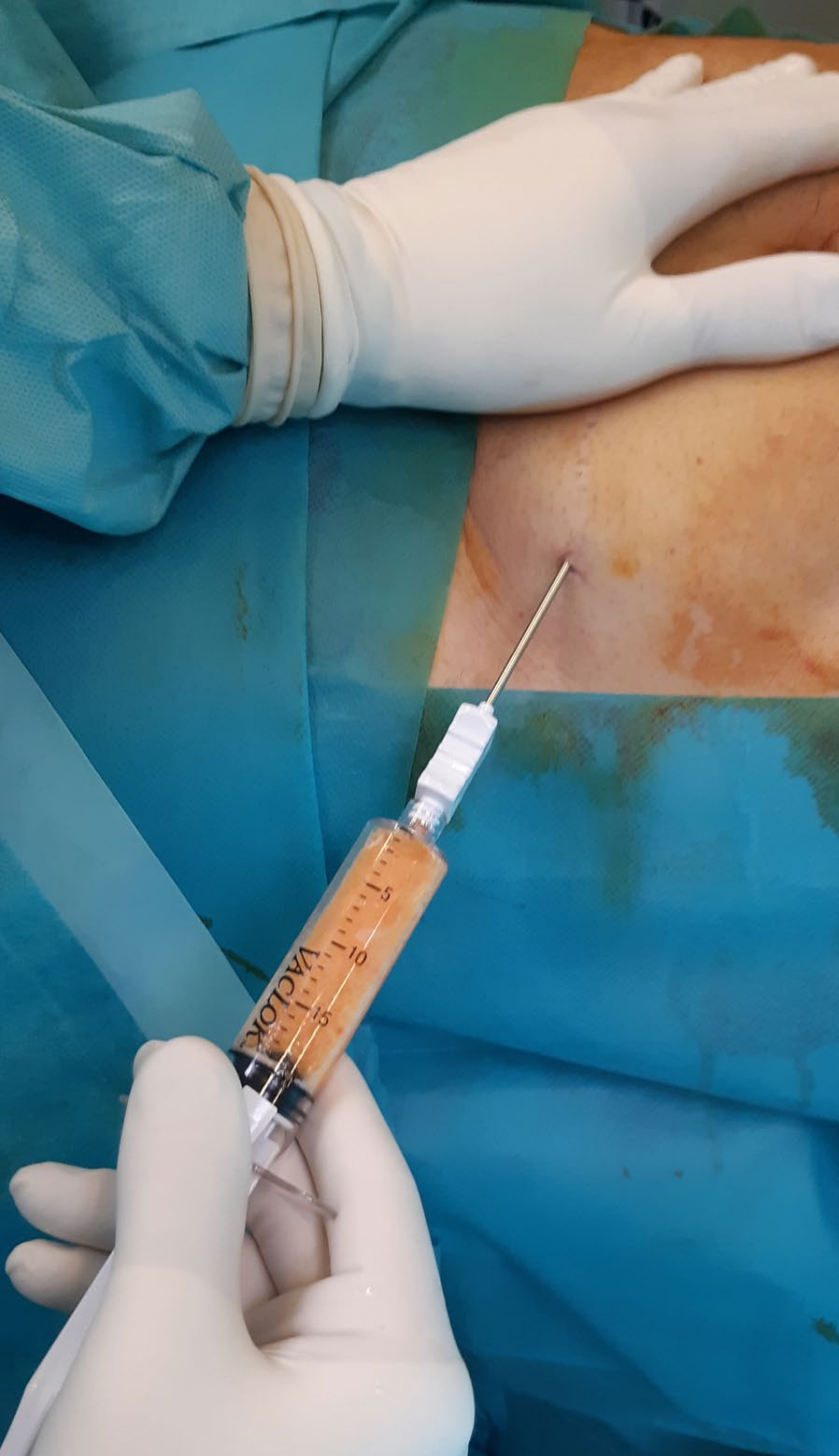
A standard percutaneous lipoaspiration is performed at least 10 min after the infiltration of the solution with ropivacaine and adrenaline; in this study, the lipoaspiration was always performed after the knee arthroscopy

**Fig. 3:**
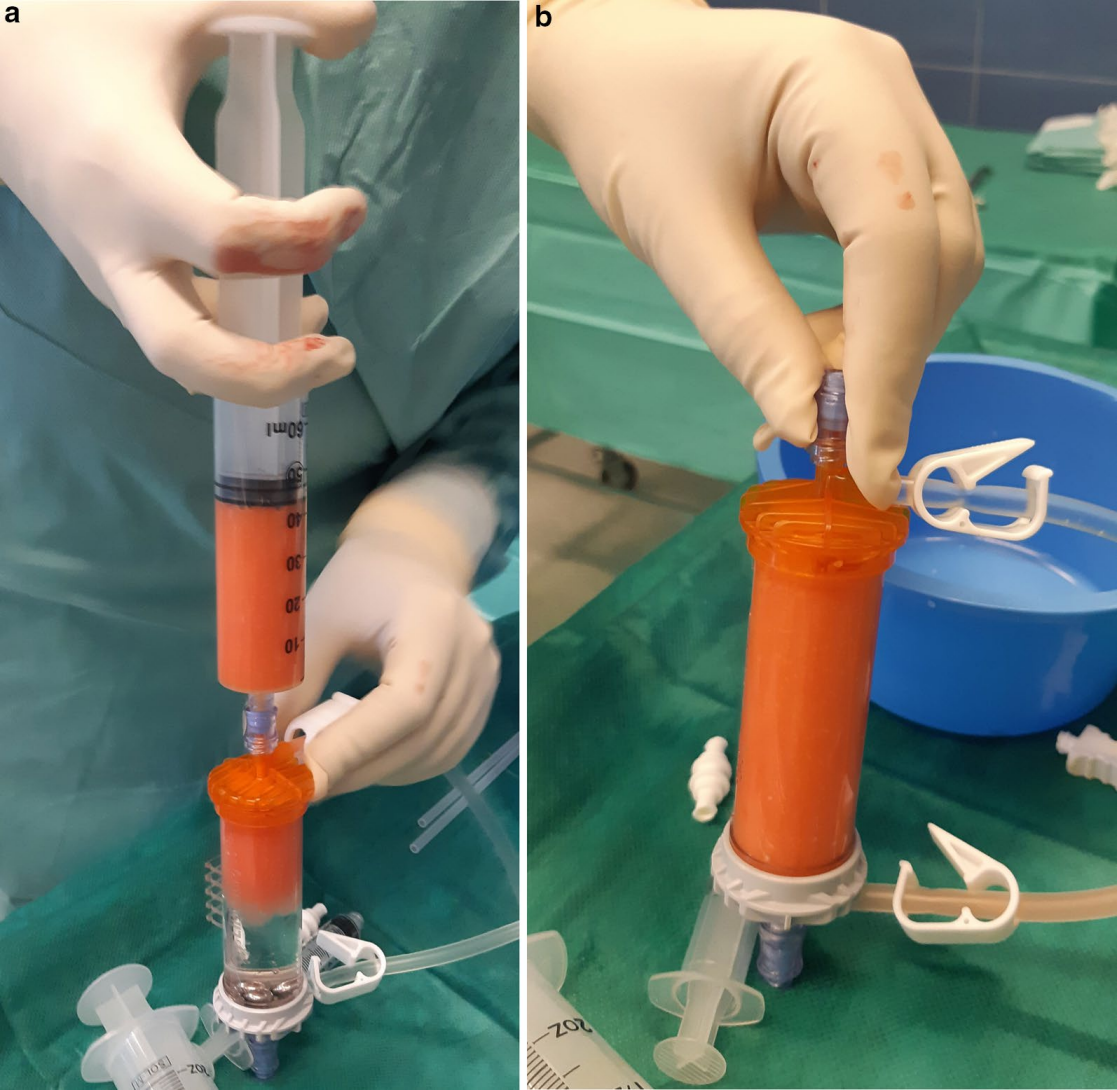
The harvested fat is transferred into the Lipogems ortho kit (**a**, **b**) to microfragment the adipose tissue (and activate the ASCs) and to wash it through a saline solution

**Fig. 4:**
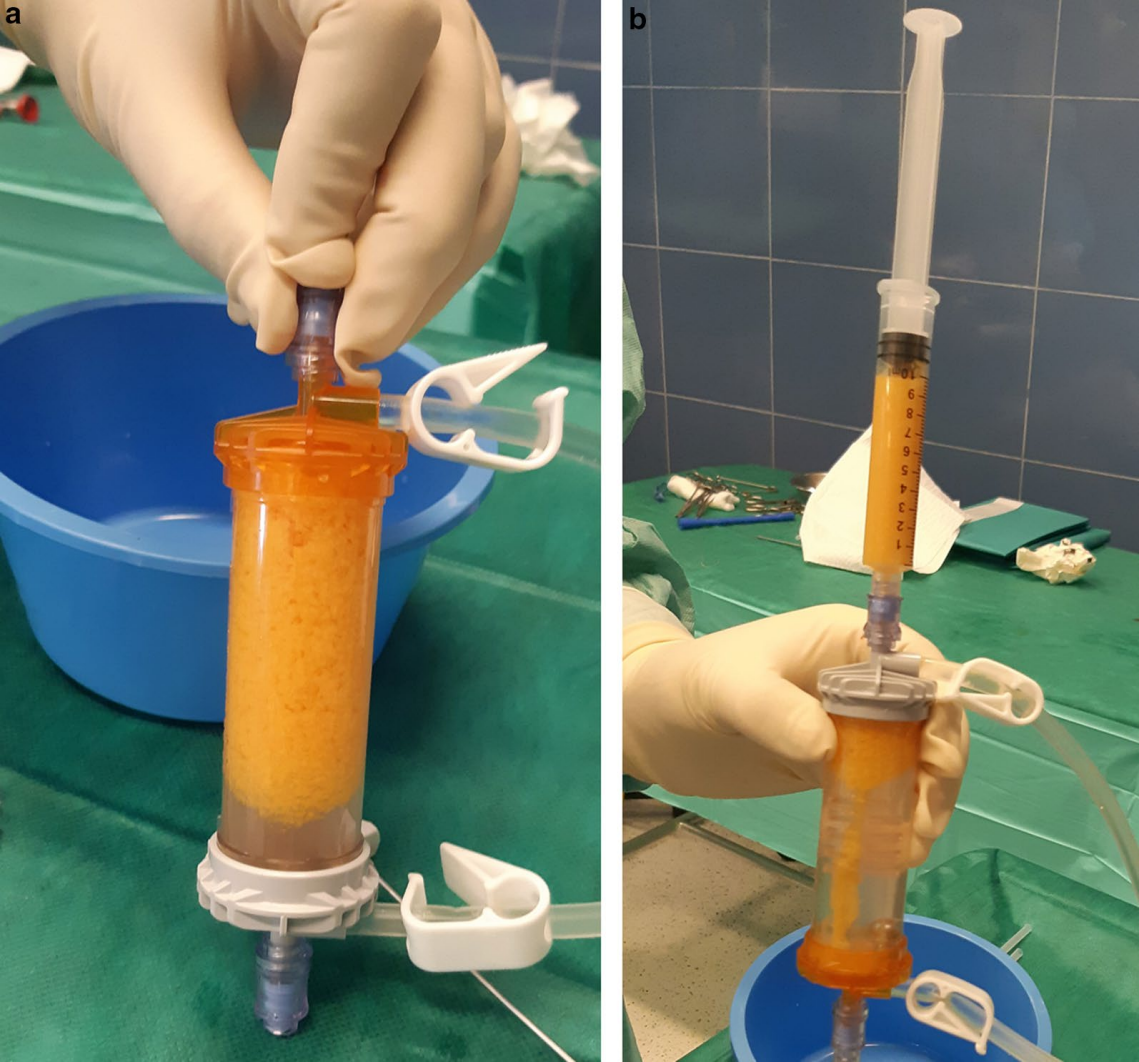
The processed final microfragmented adipose tissue product (**a**) is transferred to 10 ml syringes (**b**) for the successive intraarticular injection

**Fig. 5:**
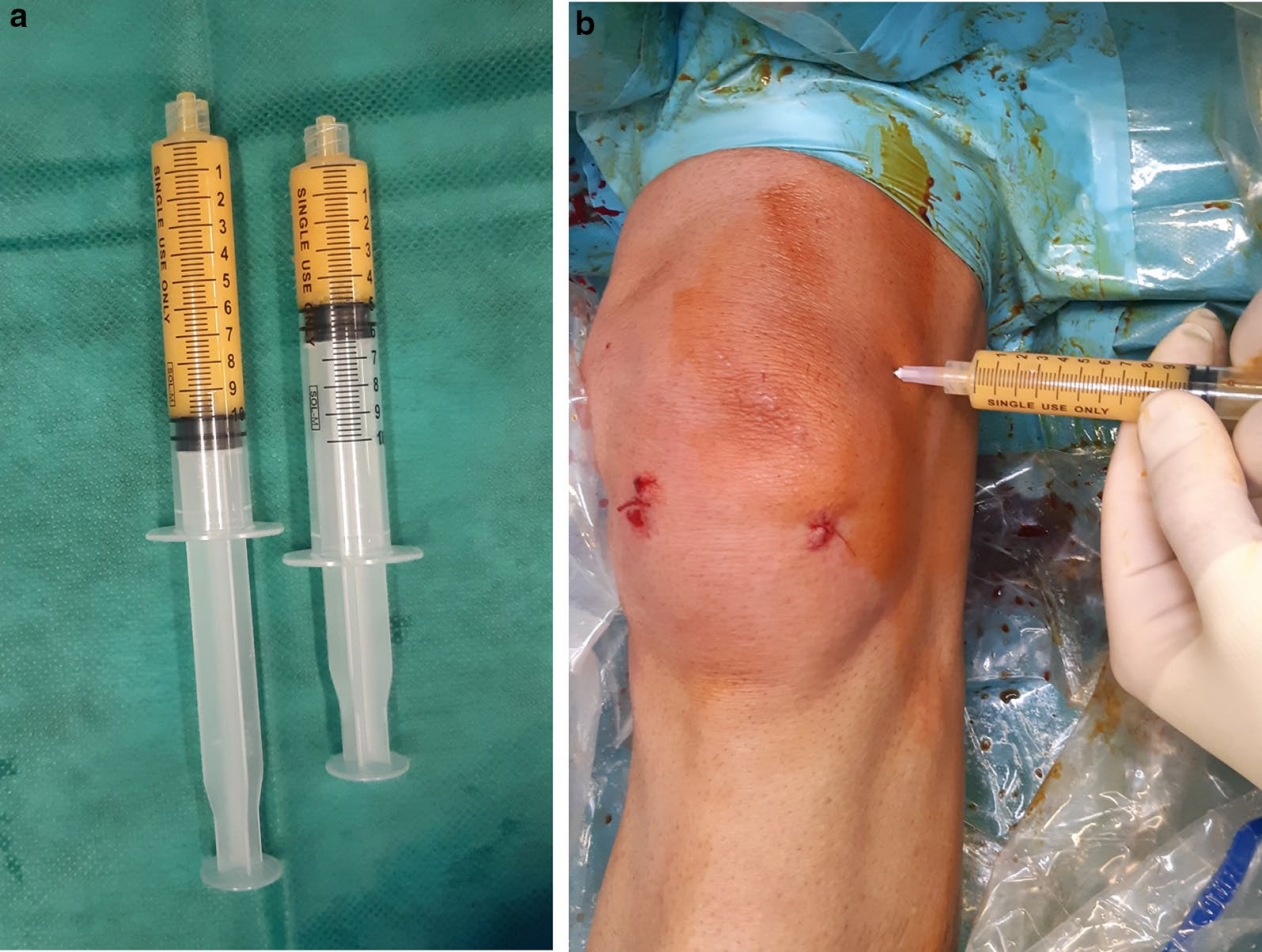
The final adipose product (10–15 ml) (**a**) is injected intraarticularly after suturing the arthroscopic portals (**b**)

The surgical procedure has been described previously [35]. Saline solution (250 ml) was mixed with two doses of 7% ropivacaine and half a dose of adrenaline, and 150–180 ml was percutaneously injected in the abdominal subcutaneous adipose tissue (Fig. 1). Before successive (at least 10 min later) adipose tissue aspiration, knee arthroscopy was performed to manage any intraarticular lesions. A standard lipoaspiration technique was then performed (Fig. 2), and the harvested fat (40–60 ml) was introduced into the Lipogems ortho kit (LIPOGEMS International SpA, Milan, Italy) (Fig. 3), according to the manufacturer’s instructions, as previously described [3]. The processed final microfragmented adipose tissue product was transferred to 10 ml syringes (Fig. 4) and injected (10–15 ml) intraarticularly after closing the knee arthroscopy portals with a 3.0 non-resorbable suture (Fig. 5).

Of the 117 patients who received this protocol, 23 patients with isolated primary PFOA were retrospectively selected and finally included in the study; the remaining 94 patients with femorotibial OA (isolated or associated to PFOA) and/or with PFOA secondary to previous patellofemoral instability or trauma were excluded. In particular, to be retrospectively included into the study, the 23 selected patients had to meet the following criteria: radiographic and (successive) arthroscopic findings of isolated primary PFOA, clinical history of prevalent anterior knee pain exacerbated by stair climbing, and absence of previous patellofemoral instability or trauma.

The 23 patients finally included into the study received only chondral shaving/abrasion and/or meniscal regularization (debridement) through the arthroscopic procedure. The postoperative regimen was the same for all 23 patients. Immediate full weight-bearing was allowed according to the feeling of each patient. Immediate knee mobilization and muscular strength exercises were started from the first postoperative day and continued for at least 3 weeks. Seven days postoperatively, all the patients were medicated and desutured. The preoperative demographic data of these patients are listed in Table 1. All those patients presented early-to-moderate (stage 1–3 according to the Iwano classification system [19]) PFOA, while none of the patients presented stage 4 (bone-on-bone) PFOA. The mean follow-up was 22.1 ± 4.2 (range, 15–30) months. Patients had been assessed preoperatively and postoperatively through the International Knee Society (IKS) knee and function scores [18] and VAS pain score. Relative to the ability to climb stairs, patients were subdivided into five groups: A: normal up and down; B: normal up, down with rail; C: up and down with rail; D: up with rail, unable down; E: unable. After surgery, patients were clinically evaluated after 3, 6, and 12 months, and every 36 months thereafter, according to personal symptomatology. Before surgery, all the patients were assessed using a detailed clinical history and an accurate physical examination; at same time, a complete radiographic study, including weight-bearing long-leg AP view radiographs, as well as Rosenberg, Merchant, and lateral projections, and magnetic resonance imaging (MRI) were performed. In general, radiographs and MRI studies were performed to diagnose and stage the OA (tibiofemoral and/or patellofemoral), and to evaluate any intraarticular lesion (ligaments, menisci, cartilage) before surgery. The ethics committee of the University of Naples (Italy) did not require approval for the review of patient records or images.

**Table 1:** Demographic data

Total number	23 patients
Age (years), mean ± SD (range)	58 ± 8 (45–78)
Gender	
Male	8 patients
Female	15 patients
PFOA stage (Iwano)	
Stage 1	5 knees
Stage 2	11 knees
Stage 3	7 knees
Side	
Right	14 knees
Left	9 knees
BMI (kg/m^2^), mean ± SD (range)	28.0 ± 4.8 (21–37)
Follow-up (months), mean ± SD (range)	22.1 ± 4.2 (15–30)

*SD* standard deviation, *PFOA* patellofemoral osteoarthrosis, *BMI* body mass index

Statistical analyses were performed using SPSS (Statistical Package for Social Sciences) version 24.0 (IBM-SPSS, New York, USA). Data were tested for normal distribution by the Kolmogorov–Smirnov *Z* test. Continuous variables were expressed using mean values ± standard deviation (SD) and range, and categorical variables were expressed as frequencies (percentages). For preoperative and postoperative comparisons of dependent variables, the paired Student’s *t*-test was used for normally distributed data. For differences in the objective stair climbing capacity, Pearson’s Chi-square test was used. Additionally, the patients were categorized into two subgroups in relation to age, BMI, and stage of PFOA, with a cutoff of 60 years of age, 30 kg/m^2^, and stage 3 Iwano classification, respectively. Differences in improvements of IKS (knee and function) and VAS scores in relation to age, BMI, and stage of PFOA were tested by unpaired Student’s *t*-tests. The level of significance was set at *p* < 0.05.

## Results

The mean IKS knee score improved from 35.6 ± 14.9 (range, 14–79) points preoperatively to 61.9 ± 17.8 (range, 27–92) points at the latest follow-up (*p* < 0.001), while the mean IKS function score improved from 52.0 ± 14.7 (range, 25–70) points preoperatively to 82.3 ± 19.1 (range, 35–100) points at the latest follow-up (*p* < 0.001). The mean VAS score decreased from 8.7 ± 2.2 (range, 0–10) preoperatively to 5.2 ± 2.5 (range, 0–6) at the latest follow-up (*p* < 0.001) (Table 2).

**Table 2:** Mean ± SD (range) of IKS knee and function and VAS scores

Value	Preoperatively	Latest follow-up	*p*-Value
Mean IKS knee (points)	35.6 ± 14.9 (14–79)	61.9 ± 17.8 (27–92)	< 0.001
Mean IKS function (points)	52.0 ± 14.7 (25–70)	82.3 ± 19.1 (35–100)	< 0.001
VAS (points)	8.7 ± 2.2 (0–10)	5.2 ± 2.5 (0–6)	< 0.001

*SD* standard deviation, *IKS* international knee scores, *VAS* visual analog scale

Paired *t*-test (*p* < 0.05)

Relative to the capacity for stair climbing, 17 (73.9%) patients were included in group C (up and down with rail) and 6 (26.1%) in group D (up with rail, unable down) in the preoperative period, while 14 (60.9%) patients were included in group A (normal up and down), 7 (30.4%) patients in group B (normal up, down with rail), and 2 (8.7%) in group C (up and down with rail) at the latest follow-up (Table 3).

**Table 3:** Capacity for climbing stairs

Group: description	Preoperatively	Latest follow-up	*p-*Value
A: normal up and down		A: 14 (60.9%)	0.03
B: normal up, down with rail		B: 7 (30.4%)	
C: up and down with rail	C: 17 (73.9%)	C: 2 (8.7%)	
D: up with rail, unable down	D: 6 (26.1%)		
E: unable			

Pearson’s chi-square test (*p* < 0.05)

No significant differences in improvements of IKS knee and function and VAS scores were found between patients of different ages (< 60 versus ≥ 60 years). Similarly, no significant differences in improvements of IKS and VAS scores were found between the other subgroups of patients: < 30 versus ≥ 30 kg/m^2^ BMI, and stage 1–2 (early) versus stage 3 (moderate) PFOA (Table 4).

**Table 4:** Differences in improvements of IKS knee and function and VAS scores relative to the age and BMI of patients and stage of PFOA

Variable (no patients)	VAS Mean ± SD	IKS knee Mean ± SD	IKS function Mean ± SD
Age ≥ 60 (9)	3.7 ± 2.1	24.3 ± 19.8	28.3 ± 14.1
Age < 60 (14)	3.4 ± 2.5	27.5 ± 20.4	31.7 ± 18.1
*p-*Value	0.50	0.80	0.46
BMI ≥ 30 (8)	3.1 ± 1.7	27.1 ± 18.8	30 ± 13.8
BMI < 30 (15)	3.8 ± 2.6	25.8 ± 20.9	30.6 ± 18.1
*p-*Value	0.14	0.55	0.70
Iwano stage 1–2 (16)	3.9 ± 2.6	29.1 ± 20.1	32.8 ± 14.9
Iwano stage 3 (7)	2.7 ± 1.4	19.7 ± 18.7	25 ± 19.5
*p-*Value	0.16	0.73	0.15

*IKS* international knee scores, *VAS* visual analog scale, *SD* standard deviation, *BMI* body mass index

Unpaired *t*-test (*p* < 0.05)

Of the 23 patients, 21 (91.3%) expressed total or near-total satisfaction with the final outcome and improvements with respect to knee function, pain, and capacity for climbing stairs. In particular, 17 (73.9%) patients reported complete or near-complete function recovery and/or pain relief, considering the procedure as excellent, and 4 (17.3%) patients achieved a satisfying function recovery and/or pain reduction, considering the procedure as good. Two (8.6%) patients of the preoperative group D (up with rail, unable down) reported a sufficient reduction of pain but were not able to recover a complete capacity to stair climbing (group C, up and down with rail) at the latest follow-up, considering the procedure as fair.

Adverse events were only related to the fat-harvesting procedure. Two patients developed a transitory hematoma of the abdominal region that did not influence the postoperative knee recovery. Neither infections nor neurovascular complications developed. A relative reduction of analgesic consumption in the postoperative period was noted, although not statistically investigated.

## Discussion

The most important finding of this study was that intraarticular injection of autologous microfragmented adipose tissue rich in ASCs following arthroscopic debridement (chondral shaving/abrasion and/or meniscal regularization) significantly and considerably increased the IKS knee and function scores and significantly decreased the VAS scores in patients with isolated primitive PFOA at a mean follow-up of almost 2 years (range, 15–30 months). The improvements in the IKS and VAS scores were not significantly affected by age, BMI, or stage of PFOA (grade 1–3). At the latest follow-up, 21 (91.3%) patients expressed satisfaction with the final outcome and improvements with regards to knee function, pain, and capacity to climb stairs, considering the procedure received as excellent (73.9% of the patients) or good (17.3% of the patients). Only two (8.6%) patients considered the procedure as fair; those patients of preoperative group D (up with rail, unable down) reported an acceptable reduction of pain and only partially improved their ability to climb stairs (group C, up and down with rail, at the latest follow-up). There were no treatment-related adverse events regarding the knee procedure. The lack of adverse events regarding the potential postoperative effusion even after injection of a high quantity of adipose tissue (10–15 ml) could be explained by the new technique of fat lavage and microfragmentation of the Lipogems ortho kit. This new system allows the accurate removal of all blood and fluids from the final product generally containing the proinflammatory molecules and cytokines, causing the possible acute inflammatory response to the adipose tissue injection reported with the previous fat preparation [1, 17, 31].

The treatment of isolated primary PFOA remains challenging, probably due to the complex etiopathogenesis of the patellofemoral pain and degeneration. Early (stage 1–2 according to Iwano classification) isolated primary PFOA results are often refractory to the conservative treatment (physiotherapy, intraarticular viscosupplementation, and/or PRP), whereas nonreplacement surgery (arthroscopic debridement with or without lateral release, chondroplasty, lateral facetectomy) results are often ineffective in patients with moderate (stage 3) isolated primary PFOA. Certainly, in all those patients, arthroplasty surgery [patellofemoral arthroplasty (PFA) or total knee arthroplasty (TKA)] appears premature and is reserved for patients with severe (stage 4) PFOA [8, 9, 33]. The 23 patients of this study presented early (70%) or moderate (30%) PFOA, whereas none of the patients presented a stage 4 (bone-on-bone) patellofemoral degeneration. In this context, the stage of PFOA was not a tool in predicting the patient outcome, as the postoperative improvements of IKS and VAS scores were not significantly correlated to the severity of PFOA; in stage 3 patients, the microfragmented fat transfer following arthroscopic debridement could also provide clinical and functional benefits. Existing literature lacks evidence on the results in patients with isolated primary PFOA treated with autologous microfragmented adipose tissue injection, focusing on results obtained in patients with generic knee OA [17, 28]. To our knowledge, only three studies have been previously published on alternative joint-preservation treatments of isolated primary PFOA: the first of these reported the results of concomitant intraarticular injection of MSCs and PRP [32], the second evaluated the efficacy and safety of autologous protein solution injection [36], the third evaluated treatment with autologous bone marrow mononuclear cells injection associated with arthroscopic debridement [14]. However, Pintat et al. [32] and Van Genechten et al. [36] showed how PFOA symptoms were recalcitrant to those treatments after 1 year of follow-up, while the present study reported good improvement at a mean follow-up of almost 2 years. On the other hand, Guimaraes et al. [14] reported the results at 2 years of follow-up, but only eight patients were treated and with different doses (range, 30–87 ml) of bone marrow mononuclear cells, while in the present study 23 patients received a standard (and recognized) dose of 10–15 ml of microfragmented adipose tissue.

As already mentioned, IKS and VAS scores were not significantly affected by age, BMI of patients, or stage of PFOA. In particular, improvements in the IKS and VAS scores were not significantly different in the various groups of patients in relation to age (< 60 versus > 60 years), BMI (< 30 versus ≥ 30 kg/m^2^), or PFOA stage (early stage 1 or 2 versus moderate stage 3). These findings suggest how the injection of microfragmented autologous adipose tissue, due to its extraordinary lubricating capacity, could be indicated both for younger patients (< 60 years) who generally present a higher functional demand, and for overweight or obese patients (in which a higher dose, 15 ml, of microfragmented adipose tissue is generally preferred). Additionally, this procedure could be beneficial in older patients (> 60 years), probably due to the fact that the concentration and the chondrogenic potential of ASCs are not strictly related to the age of the patient [5, 7]. On the other hand, the results of this study could confirm how this procedure could produce effective results in normal and underweight patients (in which the amount of lipoaspiration is often inferior resulting in not more than 10 ml of injected microfragmented adipose tissue) and in older patients, since the beneficial effect of the adipose-tissue injection is not strictly correlated to the number and concentration of the ASCs [6, 31].

It is interesting to note how, in this study, most patients significantly recovered and/or improved their ability to climb stairs: in the preoperative period, 17 patients were included in group C (up and down with rail) and 6 in group D (up with rail, unable down), while at the latest follow-up almost all (91.3%) patients were included in group A (normal up and down) or B (normal up, down with rail). On the other hand, the decrease in the VAS scores, although significant, was not conspicuous (from 8.7 points preoperatively to 5.2 points at the latest follow-up). This could be partly explained with the primary mechanical effect of the fat transfer (in terms of high articular lubrication) with consequent immediate restoration of the knee motion and function and, therefore, stair-climbing ability. On the other hand, the initial absence of the secondary biological effect of the ASCs inoculation (in terms of potential cartilage regeneration) associated to the persistence of knee synovitis due to the preexisting OA and to a possible inflammatory response to the adipose-tissue injection [1, 31] could explain the lack of conspicuous pain reduction. The surgeon could and should take into account this possibility and inform the patient preoperatively.

In our department, knee arthroscopy is always associated with the adipose-tissue injection to treat any intraarticular lesions and favor the possible future cartilage regeneration potentially induced by the ASCs [34]. In a recent meta-analysis including 18 clinical trials on the efficacy of MSCs (including ASCs, bone marrow-derived MSCs, synovial-derived MSCs, and peripheral blood-derived MSCs) in treating patients with generic knee OA, Cui et al. [22] extrapolated that patients with arthroscopic debridement and/or lower degrees of OA achieved higher outcomes. In this regard, it is important to stress again that none of the patients in this study presented a severe stage 4 PFOA. In this regard, in our department, the procedure of arthroscopy plus percutaneous fat transfer was not preferred in patients with a bone-on-bone tibiofemoral or patellofemoral OA, with the exception of patients with severe contraindications to major surgery.

The main limitation of this study was that a nonrandomized case-series was presented. Twenty-three patients with early or moderate isolated primary PFOA treated with the intraarticular injection of adipose tissue with ASCs following arthroscopic debridement were retrospectively analyzed, but results were not compared with any control group of patients treated only through an arthroscopic debridement. However, it is universally recognized that arthroscopic debridement alone (especially when performed exclusively for chondral shaving/abrasion and/or meniscal regularization without any meniscectomy or cartilage repair) is rather ineffective in improving symptoms of knee joint degeneration [13, 23]; therefore, the assumption was taken that the arthroscopic procedure in PFOA could not confound the real effectiveness of the knee injection of adipose tissue with ASCs. Similarly, one could object that in some patients, arthroscopic meniscal regularization could have improved symptoms instead of the fat transfer. All the patients that underwent meniscal regularization presented only degenerative meniscal tears in which the clinical effectiveness of the arthroscopic treatment is still highly controversial [2]. In our department, patients with recent trauma of the symptomatic knee and evident acute involvement of menisci (and/or ligaments) were originally excluded to receive this treatment protocol. Another limitation was no control group of patients treated only with adipose tissue injection without arthroscopy was included. As previously mentioned, the results of the fat transfer used as unique procedure in knee OA has already been reported to provide conflicting results in many other studies [1, 6, 22, 31]; moreover, in those studies, arthroscopic debridement has often been believed to interfere positively both with the lubricating effect of the adipose tissue and its potential capacity of stimulating the cartilage regeneration. Another limitation of this study could be that the mean follow-up (22.1 months) was quite short; however, the authors did not identify any deterioration of clinical and functional results over time, even in patients with longer follow-ups (30 months). Finally, another important limitation was that potential biological effect of the ASCs was not evaluated, neither through MRI or an arthroscopic second-look. In that regard, no patient asked for additional surgical treatment, and the authors preferred following the patients clinically and functionally, therefore carrying out only a clinical study. Similarly, the adipose tissue injected into the knee was not previously analyzed, especially concerning the number or concentration of ASCs. Anyway, it has already been widely reported how the clinical and functional results of the intraarticular injection of adipose tissue are not strictly and significantly correlated to the dose and number of ASCs they contain [21, 31].

Despite these limitations, the clinical relevance of this study is that the injection of autologous adipose tissue with ASCs associated with arthroscopic debridement is a safe and effective procedure for significantly increasing the clinical and functional scores in patients with early-to-moderate isolated primary PFOA, at a mean follow-up of almost 2 years. These results appear much more relevant when considering how the management of the first stages of the primary PFOA could be rather challenging, since conservative treatment results are often ineffective, while major surgeries or joint replacement are absolutely premature. While this study was not designed to demonstrate the biological effectiveness of the ASCs contained in the adipose tissue, nor the superiority of the adipose tissue knee injection compared with other therapies, these results failed to identify a category of patients (relative to age, BMI, or PFOA stage) who were more likely to take advantage of this protocol. Obviously, large prospective randomized clinical trials and longer follow-ups are necessary, also to evaluate the potential cartilage regeneration secondary to the advocated biologic effect derived by the inoculation of ASCs. Given the relatively high cost of this procedure, a secondary biologic effect in terms of cartilage regeneration would be strongly expected to potentially secure longer patient benefits, beyond clinical scores improvements that could be transitory.

## Conclusion

Intraarticular injection of autologous microfragmented adipose tissue rich in ASCs following knee arthroscopy for chondral shaving/abrasion and/or meniscal regularization significantly improved overall clinical and functional scores in patients with early or moderate isolated primary PFOA at a mean follow-up of almost 2 years. IKS and VAS scores were not significantly affected by age, BMI, or stage of PFOA. There were no treatment-related adverse events with regards to the knee procedure. The hope remains that the biological effect of this protocol could positively interfere with the natural history of PFOA.

## Data Availability

Not applicable.
